# Comparison of Analgesic Effect between Gabapentin and Diclofenac on Post-Operative Pain in Patients Undergoing Tonsillectomy – is the evidence enough?

**DOI:** 10.5812/atr.9879

**Published:** 2013-02-01

**Authors:** Ricardo Savaris

**Affiliations:** 1 Department of Obstetrics and Gynecology ,University of Federal do Rio Grande do Sul, Porto Alegre, Brazil

**Keywords:** Gabapentin, Diclofenac, Tonsil

*Dear Editor,*

I read with great interest the work published by Mogadam et al., entitled "Comparison of Analgesic Effect between Gabapentin and Diclofenac on Post-Operative Pain in Patients Undergoing Tonsillectomy" (1). I found the article quite interesting, however I have few questions that should be answered before we start to prescribe gabapentin or diclofenac to all patients before tonsillectomy, with the given data on pain score (Table 2), assuming that each group had an n = 30, I was not able to reproduce the significant difference among the subjects on pain scores after surgery. Calculation was done using one-way ANOVA, hence they presented their data as mean ± standard deviation. Furthermore, I believe that their results would be more meaningfull if they followed the CONSORT guidelines (2, 3). For instance, the placebo group did not receive any capsule or suppository to make the placebo effect. The authors informed that they used oral and rectal vias, and the placebo group did not receive any medication. Hence, patients were aware of what they were receiving, making the study not double-blind. Interesting enough, pain scores after surgery were the same among groups, but the placebo used a higher amount of meperidine. This could be explained the fact that the subjects were aware that they did not receive any medication for pain. Secondly, although all groups received intramuscular meperidine as needed, this drug should not be used as a pain killer. Meperidine has an erratic distribution, and its benefit as an analgesic has been questioned for long (4). Therefore, despite the interesting study undertaken by Mogadam et al. it seems that more research should be done on this subject.
